# Dilated MMA sign in cDAVF and other arterial feeders on 3D TOF MRA

**DOI:** 10.1177/19714009211041530

**Published:** 2021-08-27

**Authors:** Sin Y Foo, Saravana K Swaminathan, Timo Krings

**Affiliations:** 1 Diagnostic Neuroradiology Fellowship Program, Temerty Faculty of Medicine, Canada; 2 Interventional Neuroradiology Fellowship Program, Temerty Faculty of Medicine, Canada; 3 Division of Neuroradiology, Toronto Western Hospital (University Health Network), Canada

**Keywords:** Three-dimensional time-of-flight magnetic resonance angiography, middle meningeal artery, occipital artery, meningohypophyseal trunk, cranial dural arteriovenous fistula, ascending pharyngeal artery, posterior meningeal artery

## Abstract

**Background:**

Among the varied causes of pulsatile tinnitus, the condition that can cause severe mortality and morbidity is a cranial dural arteriovenous fistula (cDAVF). This study aimed to assess the diagnostic accuracy of the dilated middle meningeal artery on three-dimensional time-of-flight magnetic resonance angiography in cranial dural arteriovenous fistula and to identify other feeders that can aid in the detection of these lesions.

**Method:**

Magnetic resonance angiography and digital subtraction angiography data of all patients with cranial dural arteriovenous fistula treated in a single tertiary referral center between 2007–2020 were included. The middle meningeal artery and other feeders recorded from digital subtraction angiography were assessed on magnetic resonance angiography.

**Results:**

The overall agreement between readers in identifying the dilated middle meningeal artery was substantial (κ = 0.878, 95% confidence interval: 0.775–0.982). The dilated middle meningeal artery indicated the presence of a cranial dural arteriovenous fistula with a sensitivity of 79.49% (95% confidence interval: 66.81–92.16), specificity of 100% (95% confidence interval: 100.00–100.00), and negative predictive value of 94.56% (95% confidence interval: 90.89–98.02). An area under the curve of 0.8341 was observed for the ipsilateral middle meningeal artery, with a sensitivity of 92.2% and a specificity of 75.0% at a cut-off of 0.30 mm for identifying a cranial dural arteriovenous fistula. Of 73 other feeders, the occipital, meningohypophyseal trunk, ascending pharyngeal, and posterior meningeal arteries contributed to a large proportion visualized on magnetic resonance angiography (83.6% (41/49)).

**Conclusion:**

The dilated middle meningeal artery sign is useful for identifying a cranial dural arteriovenous fistula. Dilatation of the occipital and ascending pharyngeal arteries and meningohypophyseal trunk should be assessed to facilitate the detection of a cranial dural arteriovenous fistula, particularly in the transverse-sigmoid and petrous regions.

## Background

Pulsatile tinnitus is characterized by the presence of sounds such as buzzing, ringing, or whistling in one or both ears without an external stimulus, with the sounds occurring rhythmically with the cardiac cycle.^1^ An underlying cause can be identified with adequate work-up in 57–100% of the cases.^2^ The etiologies of pulsatile tinnitus are divided into two main categories: (a) vascular pathologies, including cranial dural arteriovenous fistula (cDAVF), arteriovenous malformation, aberrant course of the internal carotid artery (ICA), atherosclerosis, high-riding jugular bulb, vertebro-vertebral fistula, vascular tumors (e.g. paraganglioma), and (b) non-vascular pathologies, including idiopathic intracranial hypertension and Paget’s disease.^1,3^

A cDAVF is the most frequent cause of objective pulsatile tinnitus in patients showing normal results in otoscopic examinations^4^ and is also the condition that poses the greatest risk of mortality and morbidity without intervention, presenting with intracranial hemorrhage.^5^ Van Dijk et al.^6^ reported that the rates of intracranial hemorrhage, nonhemorrhagic neurological deficits (NHNDs), and mortality for a non-treated or partially treated cDAVF were 35%, 30%, and 45%, respectively, over a mean follow-up period of 4.3 years in patients with cortical venous drainage. cDAVFs are abnormal connections between arteries that normally feed the meninges, bones, or muscles, but not the brain and small venules within the dura mater.^7^ They are diagnosed on imaging examinations based on the presence of a hyperintense signal in dilated or non-dilated vessels on a non-enhanced time-of-flight (TOF) sequence, from retrograde leptomeningeal venous drainage and a pseudo-phlebitic pattern of the vessels.^7,8^

Magnetic resonance imaging (MRI) and magnetic resonance angiography (MRA) examinations show high diagnostic accuracy as a part of the work-up for pulsatile tinnitus.^9^ Three-dimensional time-of-flight (3D TOF) MRA is favored as a non-invasive screening tool because it has better spatial resolution and image quality and does not show venous contamination in comparison with contrast-enhanced techniques.^10^ Several studies have reported good sensitivity of 3D TOF MRA in the diagnosis of cDAVFs.^11–14^ In addition to identification of the fistulous connection, asymmetric enlargement of feeder vessels, which typically arise from the branches of the external carotid artery (ECA), has been reported as a feature for the detection of a possible cDAVFs.^15^ Being the most extensive dural feeder, the middle meningeal artery (MMA) is the most commonly involved feeding artery for cDAVFs^15,16^ and is frequently used as a pathway for transarterial embolization of cDAVF.^17^

We conducted a retrospective analysis of all cDAVFs treated at our institution that were investigated with both 3D TOF MRA and digital subtraction angiography (DSA) to assess the accuracy of the asymmetrically enlarged MMA, which we termed as the “dilated MMA” sign, in the diagnosis of cDAVFs. The other feeders documented on DSA were also analyzed on the 3D TOF MRA images.

## Methods and materials

Institutional review board approval was obtained for this single-center retrospective study at the University Hospital Network (UHN), Toronto, which is a tertiary care referral center. Informed consent was waived by the ethics committee.

### Patient selection

We searched a large local database for cases with a confirmed diagnosis of cDAVFs with the main feeders identified on DSA, the reference standard, from January 2007–July 2020. In this group, we identified patients who underwent pre-treatment intracranial MRA. Since UHN is a large tertiary referral center for treatment and management of cDAVFs, cases with MRI examinations performed at sites other than UHN were also included. We excluded cases without pre-treatment intracranial MRA or DSA data. All types of clinical presentations, that is, hemorrhagic or nonhemorrhagic presentations, were included. The inclusion criteria for the control group were pulsatile tinnitus presentation and intracranial MRA data with no underlying cDAVF on the presentation MRA or during the follow-up. A pre-existing database containing information recorded from pre-treatment DSA images was made available for characterizing the cDAVFs and assessing other feeders supplying the cDAVF.

### Imaging parameters

For patients scanned at our institution, images were acquired on 3-T scanners (GoldSeal Signa HDxt; GE Healthcare, Chicago, USA or MAGNETOM Skyra^fit^; Siemens Healthcare, Erlangen, Germany) using the 3D TOF MRA protocol (repetition time (TR) = 21 ms; echo time (TE) = 2.7 ms; field of view (FOV) = 512 × 512 mm; voxel size = 1 × 1×1 mm).

### Image analysis

#### Dilated MMA

**Figure 1.**
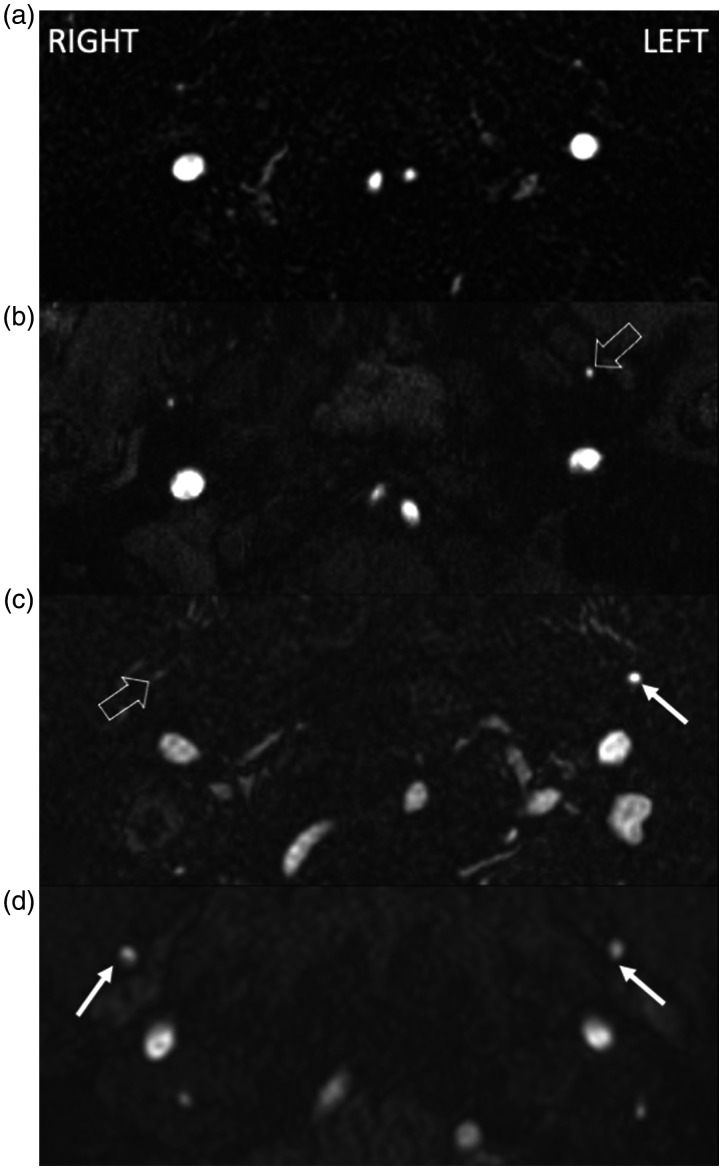
Three-dimensional (3D) time-of-flight magnetic resonance angiogram at the level of the infratemporal fossa demonstrating the middle meningeal artery (MMA) on either side. (a) No asymmetry; (b) asymmetry without a dilated MMA, more prominent on the left (open arrow); (c) asymmetry with a dilated MMA (arrow on the left, cf. white open arrow on the right); (d) bilateral dilated MMA (arrows on both sides).

For the assessment, a dilated MMA was defined as an asymmetrically enlarged MMA compared to the contralateral side (Figure 1). The MRA images in both cDAVF and control groups were de-identified and formed into a random series. Two radiologists, SYF and SKS, each with approximately 4 years of post-board certification with neuroradiology experience, analyzed the randomized series of MRA images, blinded to the results of DSA, on a PACS workstation (Coral Workstation 3.7.1.1, JDMI, UHN, Toronto, Canada). Only source data were analyzed. The extracranial and intracranial course of the MMA from its origin at the internal maxillary artery to the foramen spinosum was scrutinized. The presence or absence of a dilated MMA was visually inspected and then assigned as “Yes – unilateral or bilateral symmetrically dilated” or “No – not dilated.” Inconsistent individual assessments were re-evaluated until a consensus was reached. Quantitative measurements of both MMAs were performed using the built-in ruler tool available on the PACS system at the level of the infratemporal fossa, between the origin at the internal maxillary artery and foramen spinosum.

#### Other feeders

Targeted assessment of cDAVF feeders other than the MMA was undertaken in cDAVF patients with positive MRA findings. The feeders recorded in DSA were then visually assessed for asymmetry and dilatation in consensus by SYF and SKS. The definition of a dilated vessel was based solely on asymmetry to the contralateral side.

### Statistical analyses

Statistical analyses were performed using SAS software (version 9.4; SAS Institute, Cary, North Carolina, USA) and Excel (Microsoft, Redmond, Washington State, USA). Interobserver agreement between readers 1 and 2 for assessments of the MRA images was determined using the Cohen coefficient κ (κ ≤ 0 indicated no agreement; 0.01–0.20, none to slight agreement; 0.21–0.40, fair agreement; 0.41–0.60, moderate agreement; 0.61–0.80, substantial agreement; and 0.81–1.00, almost perfect agreement). A paired *t*-test was used to assess the MMA diameter measurements between readers.

The ability of the dilated MMA sign on MRA to predict the presence of cDAVF was calculated by determining the sensitivity, specificity, and positive and negative predictive values. Receiver operating characteristic (ROC) curve analysis was performed to determine the diagnostic performance of MMA size in differentiating patients with cDAVF from those without and differentiating patients with MMA-fed cDAVF from those with non-MMA-fed cDAVF. A value of *p* < 0.05 indicated a statistically significant result. Descriptive statistical analysis was used to represent the information for the other feeders.

## Results

A total of 1215 patients were referred to our institution for the investigation of a potential cDAVF between January 2007–July 2020. Among these, 335 patients had a confirmed diagnosis of cDAVF and were treated and managed at our institution. However, a large proportion of these patients (*n* = 285) did not undergo pre-treatment MRA and had to be excluded. Fifty-two patients underwent either 3D TOF MRA without gadolinium enhancement (*n* = 39) or contrast-enhanced MRA (*n* = 13). Finally, 39 patients who underwent both DSA and 3D TOF MRA were included. Among the remaining 880 patients who did not have a cDAVF, 279 presented with pulsatile tinnitus, of which 139 patients who underwent intracranial MRA formed the control group.

### Patient characteristics

A total of 178 patients were included in the final analysis: 39 (21.91%) with cDAVF confirmed on DSA, and 139 (78.09%) with normal reported MRA findings. The cDAVF group included 23 (58.97%) men and 16 (41.03%) women with a mean age of 57.41 ± 12.97 years. The control group included 24 (21.82%) men and 86 (78.18%) women with a mean age of 50.98 ± 15.23 years (*p* = 0.2611). Thirty (76.9%) patients with cDAVF underwent pre-treatment MRA at an external institution.

### Presentation and etiology

The initial presentation of the patients in the cDAVF group is summarized in Table 1. Seven (17.94%) patients were asymptomatic. All patients in the control group initially presented with pulsatile tinnitus.

**Table 1.** Initial presentation in the cranial dural arteriovenous fistula (cDAVF) group (*n* = 39).

Initial presentation	*n* (%)
Pulsatile tinnitus	21 (53.85%)
Hemorrhage (parenchymal and/or subarachnoid)	4 (10.26%)
Headache	3 (7.69%)
Focal neurological deficit (motor or sensory)	2 (5.12%)
Vertigo/dizziness	1 (2.56%)
Proptosis	1 (2.56%)

The final diagnoses of the patients in the non-cDAVF group are shown in Table 2.

**Table 2.** Final diagnosis in the non-cranial dural arteriovenous fistula (non-cDAVF) group (*n* = 139).

Final diagnosis	*n*
Diagnosis causing pulsatile tinnitus	15
- Carotid artery stenosis	1
- Extracranial aneurysm	2
- Jugular vein stenosis/occlusion	2
- High-riding jugular bulb	3
- Idiopathic intracranial hypertension	3
- Intracranial aneurysm	3
- Meniere’s disease	1
Unknown/no further follow-up	124^a^

^a^*n* = 3 following time-resolved MRA, *n* = 121; no further imaging.

### Characteristics of cDAVFs

The location, laterality, Borden classification, and arterial feeders of the cDAVFs are summarized in Table 3.

**Table 3.** Summary of cranial dural arteriovenous fistula (cDAVF) characteristics (*n* = 39) based on digital subtraction angiography (DSA).

cDAVF lesion characteristic	*n* (%)
Location	
Transverse-sigmoid	21 (53.85%)
Petrous	5 (12.82%)
Cavernous	2 (5.13%)
Convexity	2 (5.13%)
Tentorial – medial	1 (2.56%)
Tentorial – lateral	1 (2.56%)
Foramen magnum	1 (2.56%)
Superior sagittal sinus	2 (5.13%)
Ethmoid	1 (2.56%)
Falcine/falco-tentorial	1 (2.56%)
Hypoglossal	1 (2.56%)
Jugular	1 (2.56%)
Side of lesion	
Right	15 (38.46%)
Left	21 (53.85%)
Midline	2 (5.13%)
Bilateral	1 (2.56%)
Borden classification	
1	16 (41.03%)
2	10 (25.64%)
3	13 (13.33%)
Number of feeders	
Single feeder	3 (7.69%)
Multi-feeder	36 (92.31%)
Middle meningeal artery as feeder	
Yes	33 (84.62%)
No	6 (15.38%)

### Dilated MMA

The overall agreement between readers in identifying the dilated MMA was substantial (κ = 0.878, 95% confidence interval (CI): 0.775–0.982). There was no significant inter-reader difference in the measurements of the difference between the right and left MMAs (*p* = 0.498). Among the patients showing a dilated MMA on 3D TOF MRA (*n* = 31), all had cDAVFs (100%). A dilated MMA was missing on 3D TOF MRA in eight patients with cDAVFs and all of the patients without cDAVFs. Thus, the dilated MMA indicated the presence of a cDAVF with 79.49% sensitivity (95% CI: 66.81–92.16), 100% specificity (95% CI: 100.00–100.00), and a negative predictive value of 94.56% (95% CI: 90.89–98.02).

**Figure 2.**
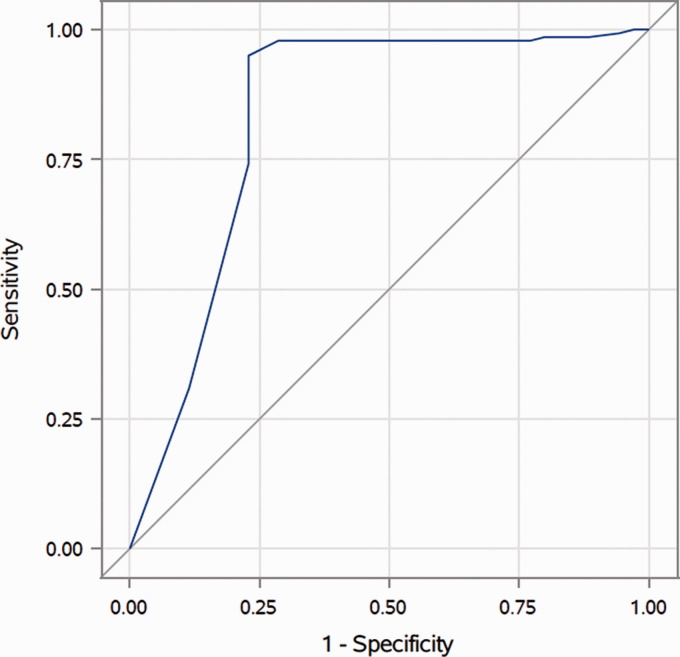
Receiver operating characteristic curve for the middle meningeal artery size on the ipsilateral side (area under the curve (AUC) = 0.8341).

Unilateral disease (*n* = 36) was selected to analyze the differences in the diameter of the MMA at the level of the infratemporal fossa. The diameter difference was statistically different (*p* < 0.0001) between those with unilateral cDAVF (0.7361 ± 0.5094 mm) and the control group (0.1295 ± 0.2080 mm). ROC analysis demonstrated an area under the curve (AUC) of 0.8341 for the ipsilateral MMA, with a sensitivity of 92.2% and a specificity of 75.0% at a cut-off of 0.30 mm for identifying a cDAVF (Figure 2).

In the cDAVF group, all patients with 3D TOF MRA findings showing a dilated MMA were confirmed to have an MMA feeder on DSA. Among those who did not demonstrate a dilated MMA on 3D TOF MRA, three had an MMA feeder on DSA, while the remaining eight did not. The dilated MMA could identify the presence of an MMA-fed cDAVF with 93.94% sensitivity (95% CI: 85.80–100.00), 100% specificity (95% CI: 100.00–100.00), and a positive predictive value of 93.94% (95% CI: 85.80–100.00).

**Figure 3.**
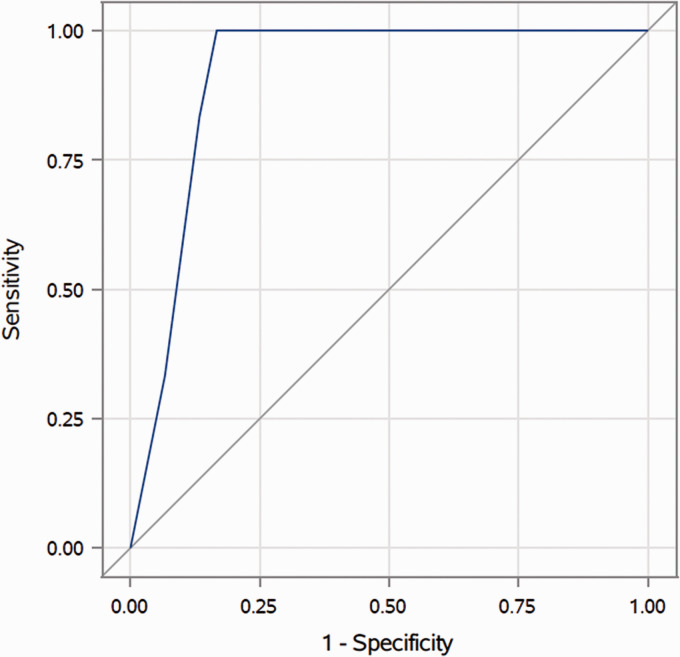
Receiver operating characteristic curve for the middle meningeal artery (MMA) size on the ipsilateral side for differentiating MMA-fed vs non-MMA-fed lesions (area under the curve (AUC)=0.9139).

Moreover, the diameter difference was statistically different (*p* = 0.0178) between patients with an MMA-fed cDAVF (*n* = 30; 0.8600  ± 0.4628 mm) and those with a non-MMA-fed cDAVF, (*n* = 6; 0.1167 ± 0.1472 mm). The AUC was 0.9139 for the ipsilateral MMA, with a sensitivity and specificity of 83.3% at a cut-off of 0.4 mm for identifying an MMA-fed cDAVF (see Figure 3).

In the three midline and bilateral lesions, the mean MMA diameter on the right was 2.567 ± 0.635 mm, and that on the left was 2.333 ± 0.602 mm (*p* = 0.7701).

### Other feeders in MMA-fed lesions

**Figure 4.**
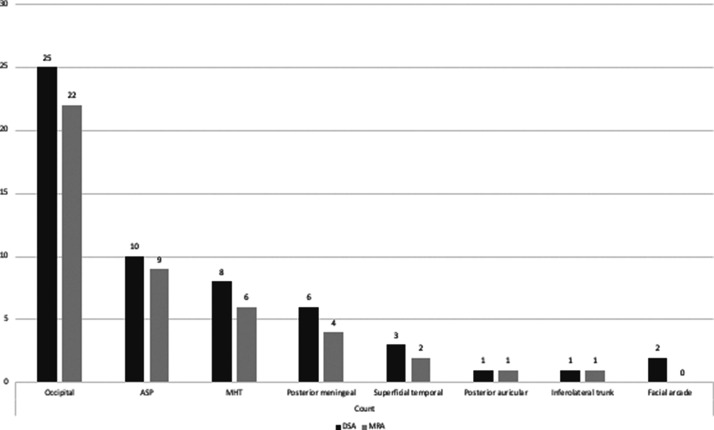
Proportions of other feeders^a^ dilated on three-dimensional (3D) time-of-flight magnetic resonance angiography (MRA) in middle meningeal artery-fed cranial dural arteriovenous fistulas (cDAVFs) in all locations. ASP: Ascending pharyngeal artery. DSA: digital subtraction angiography. MHT: Meningohypophyseal trunk. ^a^Seven other feeders appearing less frequently that are not shown in the figure are the vertebral artery muscular branch, recurrent meningeal artery (*n*=2), cerebellar pial arteries (*n*=2), caroticotympanic artery, and the Davidoff-Schechter artery.

**Figure 5.**
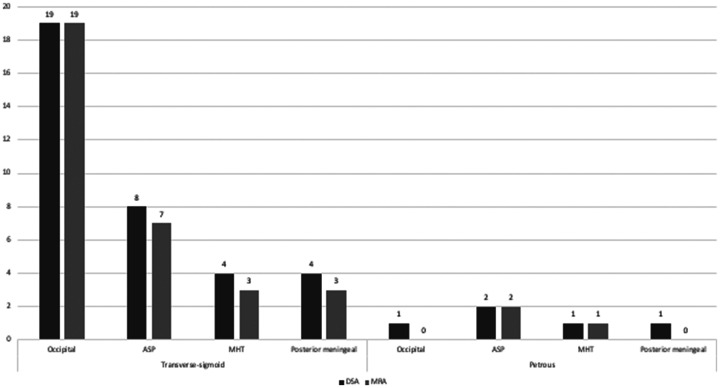
Proportions of the four main other feeders^a^ in middle meningeal artery-fed cranial dural arteriovenous fistulas (cDAVFs) visualized on three-dimensional (3D) time-of-flight magnetic resonance angiography (MRA) in the transverse-sigmoid and petrous region. ASP: Ascending pharyngeal artery. DSA: digital subtraction angiography. MHT: Meningohypophyseal trunk. ^a^Seven other feeders appearing less frequently that are not shown in the figure are the vertebral artery muscular branch, recurrent meningeal artery (*n*=2), cerebellar pial arteries (*n*=2), caroticotympanic artery, and the Davidoff-Schechter artery.

Across 31 MMA-fed cDAVFs with a dilated MMA, 73 other feeders were documented (Figures 4). Of these, 48 (65.75%) were identified as dilated compared to the contralateral side on 3D TOF MRA. When only the occipital, meningohypophyseal trunk, ascending pharyngeal, and posterior meningeal arteries were considered, the proportion visualized on MRA increased to 83.6% (41/49) (Figure 5).

### Non-MMA-fed lesions

Six (15.38%) lesions were not fed by the MMA, and two of these had a single feeder. Two were located at the transverse-sigmoid and one each in the cavernous, hypoglossal, ethmoid, and jugular regions. All feeders (both single- and multi-fed, *n* = 12) were analyzed simultaneously. Four of these were visualized as dilated on 3D TOF MRA. These were the ascending pharyngeal artery (*n* = 2), cavernous ICA (*n* = 1), and ethmoidal artery (*n* = 1). The proportion of all feeders observed as dilated on MRA in MMA-fed lesions was 59.31%, compared to 38.10% in non-MMA-fed lesions (*p* = 0.5872).

## Discussion

cDAVF can have benign and aggressive presentations. When the presentation is aggressive, the diagnosis is based on a “pseudo-phlebetic” pattern in structural imaging, venous congestion, and hemorrhage. The benign subtype can present with pulsatile tinnitus, although the most common presenting symptom, particularly in the transverse-sigmoid region,^5^ is nonspecific due to the aforementioned wide differential diagnoses. The work-up of patients with pulsatile tinnitus involves structural and vascular imaging. 3D TOF MRA has been shown to be a useful screening tool in the detection and follow-up of vascular anomalies, including arteriovenous malformations and aneurysms. A few published studies have proven the efficacy of 3D TOF MRA in the detection of cDAVFs.^14,15^ Various features of vascular imaging have been described, including flow signal in the draining veins, angioarchitecture of the fistulous connection, asymmetry and enlargement of feeder arteries,^18^ and demonstration of cortical venous reflux. Other techniques have also shown good sensitivity and specificity,^13,18^ although they have drawbacks. Computed tomography angiography (CTA) requires contrast administration and radiation exposure. Time-resolved methods, on the other hand, are highly specialized imaging techniques that are of limited use in non-neurospecialist centers.^13,19,20^

In this study, the dilated MMA sign was shown to have good sensitivity and high specificity for the detection of a cDAVF lesion, as its diameter appeared to be significantly increased relative to those seen in MRA of patients without cDAVF. The lesions were mainly distributed in the transverse-sigmoid and petrous regions, where four other feeders were identified as relatively important contributors to MMA-fed cDAVFs.

The MMA is the most commonly cited arterial feeder of a cDAVF, and is also the main route for transarterial embolization of cDAVF lesions.^21^ In our analysis, the dilated MMA sign showed 79.49% sensitivity and 100% specificity in detecting the presence of a cDAVF. An ROC analysis demonstrated an AUC of 0.8341 with a difference as small as 0.3 mm between dilated and non-dilated MMA, allowing differentiation between patients with and without the lesion with 91.4% sensitivity and 77.1% specificity. These findings are comparable to those reported in a recent study by Lee et al.,^15^ which found that 14 of 17 MMA-fed lesions showed enlarged MMAs. The overall agreement between readers in identifying the dilated MMA sign was very good (κ = 0.878), slightly better than the previously reported value in a similar study by Azuma et al.^14^ (κ = 0.711), which assessed the main arterial feeders on 3D TOF MRA. These findings emphasize the use of the dilated MMA sign as a clue for the diagnosis of cDAVFs.

The MMA is one of the most extensive branches of the ECA and supplies more than two-thirds of the cranial dura, making it one of the most important dural arteries.^22^ The classic origin of the MMA is from the internal maxillary artery (IMA), but it has been also reported to originate from the basilar artery, occipital artery, and cavernous ICA.^23^ The MMA can rarely arise from the ophthalmic artery instead of the IMA (incidence, 0.5%),^24^ resulting in an apparently enlarged contralateral MMA at the level of the infratemporal fossa. This was observed in three cases in our control group. The origin of the MMA should be assessed to avoid false-positive findings on the contralateral side.

Our study is one of the few with a large series that systematically demonstrated dural arterial feeders on MRA. The MRA images were presented to observers with a start and end slice number covering only the region of the infratemporal fossa to minimize the potential for observers to identify any other signs of cDAVF in the remainder of the scanned volume. Among the 73 feeders that were assessed, 48 (65.75%) were identified as dilated or asymmetrically enlarged. These were further stratified by location, with transverse-sigmoid and petrous locations being the two most common locations in our cohort. The study by Lee et al.^15^ (*n* = 19) is the only other study documenting asymmetric enlargement of other feeders, namely the branches of the ECA, on 3D TOF MRA. Lee et al. pre-determined the branches of the ECA i.e., the ascending pharyngeal artery, middle meningeal artery, accessory meningeal artery, artery of the foramen rotundum, and the occipital artery) to be analyzed. In our cursory assessment of all feeders demonstrated on DSA, we additionally identified the meningohypophyseal trunk and posterior meningeal artery as potentially prominent on 3D TOF MRA, thereby suggesting that these vessels should also be scrutinized. Most other studies assessed the feeders using techniques other than MRA, including ultrasound, CTA, and pseudo-continuous arterial spin-labeling.^18,25–27^

**Figure 6.**
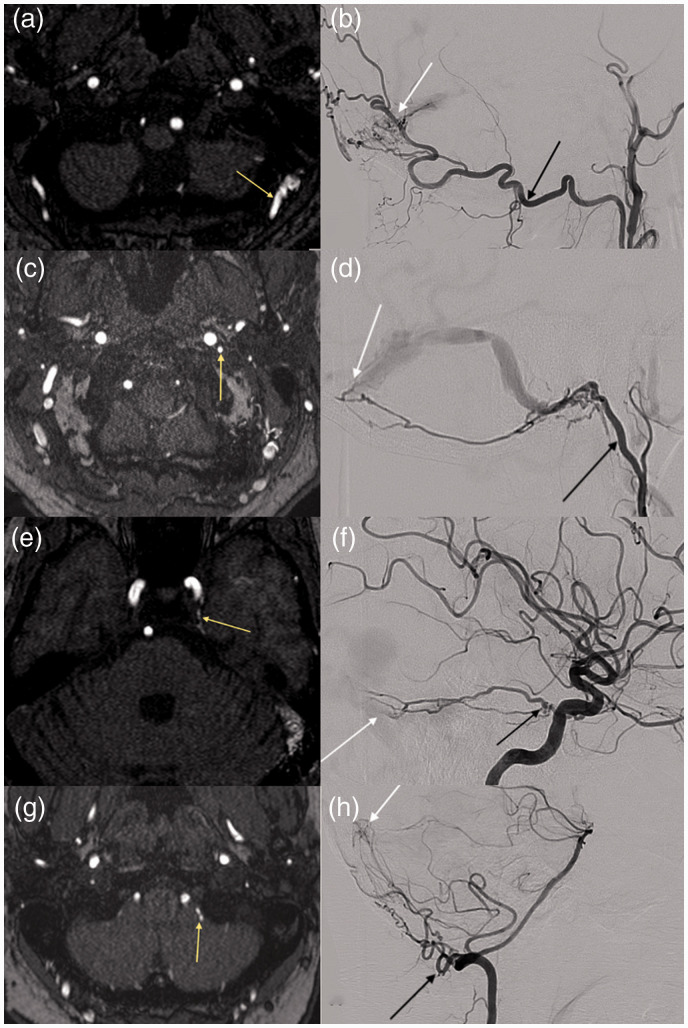
Examples of the four main other feeders observed as dilated on magnetic resonance angiography (MRA) with the corresponding digital subtraction angiography (DSA) images. (a), (c), (e), and (g): Axial three-dimensional (3D) time-of-flight MRA. (b), (d), (f), (h): DSA. Yellow arrows denote dilated vessels on axial three-dimensional (3D) time-of-flight MRA images, black arrows denote the corresponding arterial feeder on DSA supplying the fistula (white arrows). (a) Dilated left occipital artery. (b) Lateral projection and injection of the left external carotid artery (ECA) showing the occipital artery supplying a transverse-sigmoid cranial dural arteriovenous fistula (cDAVF) in the same patient. (c) Dilated left ascending pharyngeal artery. (d) Antero-posterior projection and injection of the left ECA shows the ascending artery supplying a transverse-sigmoid cDAVF in the same patient. (e) Dilated left meningohypophyseal trunk. (f) Lateral projection and injection of the left internal carotid artery showing the meningohypophyseal trunk supplying a transverse-sigmoid cDAVF in the same patient. (g) Dilated left posterior meningeal artery. (h) Lateral projection and injection of the left vertebral artery showing the posterior meningeal artery supplying a transverse-sigmoid cDAVF.

The occipital, ascending pharyngeal, meningohypophyseal trunk, and posterior meningeal arteries represent the majority of other feeders in MMA-fed lesions (*n* = 32). Collectively, 83.67% (41/49) of these were deemed dilated on MRA. Figure 6 shows examples of vessels dilated on MRA with the corresponding DSA. The anastomoses formed between the occipital and ascending pharyngeal arteries with the branches of the MMA were the most likely explanation: the mastoid branch of the OA with the petrosquamous branch of the MMA and carotid and jugular ascending pharyngeal branches with the petrosal and petrosquamous MMA branches, respectively.^23^ In the case of a dilated occipital artery, Hisaeda et al.^26^ found similar results when assessing this vessel in transverse-sigmoid lesions on ultrasound.

Cavernous cDAVF lesions are typically the second-most common,^28^ and Bing et al.^29^ demonstrated that these lesions are frequently (16/23, 69.56%) fed by the ascending pharyngeal feeder. The lack of cavernous lesions in our study (*n* = 2) likely explains the smaller number of ascending pharyngeal feeders in our cohort, which is less than half of the number of occipital feeders. This reflects the selection bias resulting from analyzing the MRA examinations only. Cavernous sinus lesions usually present with orbital symptoms such as chemosis, ophthalmoplegia, and proptosis,^30^ and the primary investigation is with CT angiogram.

The meningohypophyseal trunk was observed in all cases at the petrous (*n* = 2, 100%) and occasionally at the transverse-sigmoid lesions (*n* = 3, 75%) on MRA. The meningohypophyseal trunk is not usually discernible under normal circumstances on a 1.5-T system, but can be a normal finding on a 3T system.^31^ It should still be evaluated because one of its main branches, the lateral tentorial artery, has anastomoses with the petrosquamous branch of the MMA and occipital artery as it runs along the lateral edge of the tentorium, connecting the petrous to the transverse-sigmoid junction,^31^ potentially co-feeding an MMA-fed cDAVF.

A posterior meningeal feeder was present in only six cases (15.38%), despite this artery being one of the main three suppliers to the supra-tentorial dura, which also has anastomoses with the MMA.^23^ A possible explanation would be that the dural territory supplied, namely the convexity (parieto-occipital) and superior sinus, was represented in small numbers in our group of lesions (*n* = 3 and *n* = 2, respectively). Due to its location, arising from the third segment of the vertebral artery, it typically supplies cDAVF of the tentorium, torcular, transverse-sigmoid sinus, or cervicomedullary junction.^32^ Of six cDAVFs with a posterior meningeal feeder, four were located at the transverse-sigmoid sinus, one at the petrous region, and one over the convexity. Only four (66.67%) were dilated on MRA. This is likely limited by the smaller vessel diameter to the MRA image voxel size ratio and the orientation of the vessel relative to the scan plane.^14,32^

There were two cases in which the MMA was a feeder on DSA, however, no dilated MMA was observed on MRA. In one case, the lesion was singly fed by the MMA at the right convexity (Borden 3), and in the other, the lesion was at a cavernous location (Borden 2), additionally fed by the inferolateral trunk and artery of the foramen rotundum. The inferolateral trunk was dilated on MRA. Both lesions had small shunts from the MMA on the DSA.

Fistulous connections or features of venous congestion and cortical venous reflux may not always be demonstrated on structural or vascular imaging, particularly in the benign subtype. Our study highlights the importance of scrutinizing a dilated MMA, which has been shown to have good sensitivity and specificity in detecting cDAVF. Furthermore, our analysis characterized the presence of a few other dural feeders that could potentially be helpful in increasing the confidence of diagnosing a cDAVF, particularly in the transverse-sigmoid and petrous regions in patients with non-severe symptoms.

We did not analyze the relationship between the dilated MMA sign and other imaging features of cDAVF lesions. The association with other parenchymal findings is important because natural history studies of cDAVF suggest that although a benign presentation such as pulsatile tinnitus portends a better prognosis, there remains a risk of conversion to an aggressive subtype.^30^ Future studies are required to assess this relationship. Furthermore, two cases highlighted the effect of the shunt size on the visibility of a feeder vessel; therefore, investigation of the signal intensity within a vessel as another feature signifying the role as a feeder is required since flow velocities influence this finding on 3D TOF MRA.

The small sample of non-MMA-fed cDAVF lesions in our group precludes meaningful analysis. Although the difference was not statistically significant, a lower proportion of feeders was visibly dilated on MRA in non-MMA-fed lesions compared to those in MMA-fed lesions. This could hint at the notion that flow hemodynamics of a dural AV shunt is increased when the MMA is involved, likely due to its large anastomotic network and anatomical and embryological contribution to the meningeal vascular supply.

The proportion of women with pulsatile tinnitus in the control group was three-fold that of men. Pulsatile tinnitus commonly affects overweight women in the third to the sixth decade and can be a presentation for intracranial hypertension. A review by McGeeney and Friedman^34^ proposed that aldosterone and vitamin A, which is influenced by estrogen and adipose tissue, contribute to the pathophysiology of pseudotumor cerebri, a.k.a. idiopathic intracranial hypertension. Although the exact proportion of women in the control group who were overweight is not known, this theory speculates that women can be more sensitive to alterations in intracranial pressures, which in turn causes turbulent intracranial blood flow transmitting through the auditory structures, resulting in pulsatile tinnitus.

### Study limitations

The primary limitation of the study was that it was a retrospective analysis, which introduced an inherent propensity for bias. Second, information on the other feeders was obtained from a pre-existing database in which up to four feeders mentioned in the DSA report were recorded without any order of importance, and the smaller shunts that do not demonstrate vessel dilatation on MRA were not taken into account. As a result, a small group of feeders was excluded from the analysis of five cases. However, given the skew of the data toward other feeders in the transverse-sigmoid and petrous lesions, the authors deemed it acceptable for the purposes of this review. Third, the other feeders were not objectively assessed in the control group to confirm that these were definitely not dilated. Therefore, the presence of meningohypophyseal trunk and posterior meningeal artery dilatation cannot be readily generalized. Nevertheless, these two vessels were additionally seen as dilated on 3D TOF MRA and so could potentially be important feeders. Further studies are required. Fourth, the heterogeneity of cDAVF in a small sample size (n = 39) precludes meaningful statistical analysis. Finally, 121 (87.0%) patients in the control group did not have a final diagnosis and lacked DSA data. Even with thorough interrogation of the regional electronic medical records and a mean time interval of 4 years between the MRA examination and the date of manuscript submission (range: 8 months to 13 years), the risk of overlooking small cDAVF lesions could not be eliminated. However, this scenario represents real-life clinical practice, because a patient would not necessarily have undergone DSA, which carries a small but significant risk of permanent neurological damage,^35^ when the only clinical presentation is pulsatile tinnitus.

## Conclusion

In pulsatile tinnitus, dural AV fistulas most commonly show a transverse-sigmoid location. In our analysis, the MMA was by far the most common feeding vessel. Considering the significant difference in diameter between normal and abnormal MMAs in the lesion and control groups, for pulsatile tinnitus, the dilated MMA sign is useful for identifying a dural AV fistula. In addition to the MMA, dilatation of the occipital artery and ascending pharyngeal and meningohypophyseal trunk should be scrutinized to increase the likelihood of detecting a cDAVF, particularly for lesions in the transverse-sigmoid and petrous regions. Knowledge of the branches and anatomy of the MMA is very useful when evaluating the meningeal vessels to exclude a cDAVF. cDAVFs in other locations show different presentations such as exophthalmos and chemosis in cavernous lesions and intracranial hemorrhage in ethmoidal fistulas. The role and involvement of the MMA in these other locations is unclear, and further studies are required.
